# Under-Reporting of Adverse Drug Reactions in Finland and Healthcare Professionals’ Perspectives on How to Improve Reporting

**DOI:** 10.3390/healthcare10061015

**Published:** 2022-05-31

**Authors:** Andreas Sandberg, Veera Salminen, Susanna Heinonen, Mia Sivén

**Affiliations:** 1 Division of Pharmaceutical Chemistry and Technology, Faculty of Pharmacy, University of Helsinki, FI-00014 Helsinki, Finland; veera.salminen@helsinki.fi (V.S.); mia.siven@helsinki.fi (M.S.); 2 Biocodex Oy, FI-02101 Espoo, Finland; s.heinonen@biocodex.fi

**Keywords:** adverse drug reaction reporting, pharmacovigilance, follow-up information

## Abstract

Background: Adverse drug reaction (ADR) reporting has been studied relatively extensively in all the Nordic countries besides Finland, but no definitive solution to decrease under-reporting has been found. Despite many similarities in reporting, the most notable difference compared to other Nordic countries is that ADR reporting is completely voluntary in Finland. Purpose: The purpose was to examine if voluntary reporting influences healthcare professional (HCP) ADR reporting, why HCPs do not report all suspected ADRs, how could reporting be enhanced, and do we need to develop the process for collecting ADR follow-up (F/U) information from HCPs. Methods: An open and anonymous questionnaire was developed and made available online at the e-form portal of the University of Helsinki. Trade and area unions distributed the questionnaire to their respective member physicians, nurses, and pharmacists. Two independent coders performed the content analysis of answers to open-ended questions. Results: A total of 149 responses was received. Two fifths (38%) of the HCPs confirmed that they had not always reported suspected ADRs. The main reason for not reporting was that the ADR was already known. HCPs who had no previous ADR reporting experience did not report ADRs mainly because it was not clear how to report them. Seriousness (chosen by 76%) and unexpectedness of the reaction (chosen by 64%) were the most actuating factors in reporting an ADR. Only 52% of the HCPs had received ADR reporting training and only 16% of the HCPs felt that they had enough information about reporting. Most HCPs felt that ADR F/U requests are justified, and these requests did not affect their ADR reporting willingness. Conclusions: As in other Nordic countries, ADR under-reporting occurs also in Finland despite differences in reporting guidance. ADR reporting rate could be enhanced by organizing recurring training, information campaigns, and including reporting reminders to the patient information systems that HCPs use. Training should primarily aid in recognizing ADRs, educate in how to report, and promote a reporting culture among HCPs.

## 1. Introduction

At the time of the marketing authorization (MA) approval, the approved medicines often only have safety data about a limited patient population in controlled experimental conditions [1,2,3,4]. During the post-authorisation period, the patient population expands significantly, and treatment conditions become much more diverse. Therefore, the safety profile of approved medicines needs to be continuously monitored. Collecting spontaneous adverse drug reaction (ADR) reports is the main source of safety information during the post-authorization period, although under-reporting of ADRs is generally recognized [4,5,6,7,8,9,10,11].

In Finland, research focusing on ADR reporting is scarce, and especially ADR under-reporting has not been studied at all. In other Nordic countries, ADR reporting has been studied relatively extensively. Despite the similarities between these Nordic, European Economic Area (EEA) countries, there are some national differences in ADR reporting guidance, obligations, and routes [12,13,14,15,16,17,18,19,20,21]. For Finland, the most notable difference compared to other Nordic countries is that ADR reporting is completely voluntary for all healthcare professionals (HCPs) [12,13,14,15,16,17,18,19,20,21]. In the EEA, HCPs and patients can report suspected ADRs to the pharmaceutical company or to the national competent authority, which then forwards the information to the EudraVigilance-database, which is operated by the European Medicines Agency (EMA) [4,22].

Although ADR under-reporting has been studied in other Nordic countries during the last 30 years, no definitive solution to decrease under-reporting has been found. According to these studies, the under-reporting rate has remained high (75–99%) during this time span [9,23,24,25]. A survey carried out in an environment where reporting is voluntary could bring valuable new insights into the research field.

Reasons for under-reporting and factors affecting reporting willingness have been studied especially in Sweden. Familiarity with the ADR encountered, not remembering to report, and lack of time were the main reasons why physicians did not report suspected ADRs [26,27]. Familiarity with the ADR encountered was also the main reason for nurses, but lack of knowledge as to how to report and what to report were the next most prevalent reasons not to report ADRs [28]. Correspondingly, for both physicians and nurses the most important factors affecting willingness to report were the severity and unexpectedness of the ADR in addition to ADRs occurring with new medicines [27,28]. It is noteworthy that ADR reporting of pharmacists has barely been studied in the Nordic countries. A single Norwegian study concluded that pharmacists considered lack of time, confidence, and knowledge about reporting to be factors that could make them refrain from reporting ADRs [29].

We carried out a cross-sectional survey of HCPs (i.e., physicians, pharmacists, and nurses) to discover how extensive under-reporting is in Finland, why HCPs do not report all suspected ADRs, how could reporting be enhanced, and do we need to develop the process for collecting follow-up (F/U) information from HCPs. This survey follows our first research, which focused on additional monitoring awareness in Finland [30].

## 2. Methods

### 2.1. Questionnaire Design

An open and anonymous questionnaire was developed and made available online at the e-form portal of University of Helsinki. The authors determined the final wording of the questionnaire. The questionnaire consisted of a cover letter including informed consent statement and a maximum of 25 questions. The number of questions varied from 18 to 25, depending on respondents’ answers. Six questions were open-ended. The questionnaire is presented in the Supporting Information section of this article.

The face validity of the questionnaire was tested in a small-scale pilot study with eight HCPs (two M.Sc. pharmacists, two B.Sc. pharmacists, one nurse, one physician, and two dentists). Pilot study participants represented the Finnish healthcare system well as their primary workplaces included a hospital, a healthcare center, a retail pharmacy, and a private clinic.

In Finland, pharmacists licensed to practice the profession are Bachelors of Science (B.Sc.) in Pharmacy (1st cycle degree) or Masters of Science (M.Sc.) in Pharmacy (2nd cycle degree) graduates [31]. Both groups work with patients and have similar responsibilities in the patient interface. Physicians are primarily responsible for prescribing medicines in Finland [32]. In-service trained nurses are also allowed to prescribe a restricted set of medicines [32]. There are approximately 30,000 physicians, 6000 pharmacists, and 165,000 nurses in Finland [33,34,35].

Based on the pilot study, small modifications were made to the questionnaire. Due to the modifications, the results of the pilot study were not included in the final results of this research. No problems were observed with the e-form portal. It was estimated that answering the questionnaire would take 10–20 min.

### 2.2. Questionnaire Distribution

A convenience sample was collected by requesting trade and area unions to invite their respective member physicians, nurses, and pharmacists to complete the questionnaire. The invitation and link to the questionnaire were sent to the HCPs via email or by attaching it to a union newsletter. When applicable, a reminder was sent to maximize the number of responses. Finnish Medical Network (www.fimnet.fi) was also utilized in questionnaire distribution by adding an invitation to complete the survey to their HCP restricted front page. HCPs working for the government or pharmaceutical industry were excluded as they do not primarily work with patients and ADR reporting. No honorarium was provided to the respondents. Answers were collected during the COVID-19 pandemic, from March 2021 to May 2021.

### 2.3. Analysis

#### 2.3.1. Statistical Analysis

GraphPad Prism Version 9.1.0 (GraphPad Software, San Diego, CA, USA) was used to analyse the data. Chi-square test for independence was used as the primary analysis method. Fisher’s exact test was used to confirm the exact *p*-value when applicable. A 5% significance level applies in all hypothesis testing.

#### 2.3.2. Content Analysis

Two independent coders familiar with adverse event reporting carried out content analysis of answers to open-ended questions. Both inductive and deductive approaches were applied as the approaches are not mutually exclusive [36]. Reclassification of answer categories was performed in situations where the independent coders had initially created differing categories. Any differences in the classifications were discussed between the two coders until a consensus was reached. EMA guidance and an important medical event (IME) terms list were utilized in the classifications, where applicable [37].

## 3. Results

A total of 149 responses were received. Trade and area unions did not reveal the exact number of members they had in their organizations, which made response rate calculations impossible. Five responses did not meet the inclusion criteria for the profession (i.e., physician, pharmacist, or nurse) and seven for the working place (government and pharmaceutical industry excluded). In addition, five responses had to be excluded as the respondents stated having technical difficulties when answering the questionnaire. A total of 132 responses were analyzed (78 B.Sc. pharmacist, 21 M.Sc. pharmacist, 20 physician, and 13 nurse).

### 3.1. Demographics

HCPs answering the questionnaire were professionally experienced. The majority (77%, *n* = 102) had a minimum of 10 years of experience in their profession. Most experienced were the physicians as 90% (*n* = 18) of them had at least a decade of experience.

The primary workplace was a healthcare center for most physicians (45%, *n* = 9) and nurses (69%, *n* = 9). The retail pharmacy was the primary workplace for most B.Sc. pharmacists (83%, *n* = 65) and M.Sc. pharmacists (67%, *n* = 14). The demographics are summarized in Table 1.

### 3.2. Adverse Drug Reaction Reporting Experience and Knowledge

**Figure 1:**
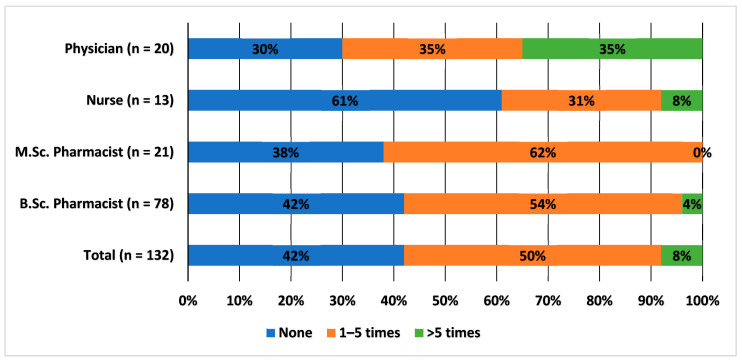
Adverse drug reaction reporting experience among healthcare professionals, percentage (%) of HCPs. Questionnaire question: During your career, how many times have you reported a suspected adverse drug reaction to the local health authority (Finnish Medicines Agency) or the marketing authorization holder?

Two fifths (42%, *n* = 55) of the HCPs responding to the questionnaire had not reported any ADRs during their careers and half (*n* = 66) of the HCPs had reported an ADR one to five times. Only 8% (*n* = 11) had reported an ADR over five times. Interestingly, the nurse group was the only group in which over half of the respondents had never reported an ADR (61%, *n* = 8). The physician group was the only group in which a significant portion of the respondents had reported an ADR over five times (35%, *n* = 7). The ADR reporting experience is presented in Figure 1.

Of all the respondents, 40% (*n* = 53) believed erroneously that reporting is obligatory for HCPs in Finland. Only 17% (*n* = 23) knew that reporting is not obligatory whereas 21% (*n* = 28) stated that they did not know the answer. The rest of the respondents (21%, *n* = 28) thought that it is only obligatory for medicines under additional monitoring or for vaccines.

HCPs also responded to an open-ended question regarding their thoughts as to what kind of ADRs should be reported. The two most common answer categories recognized were “Unknown/Unexpected ADRs” (44%, *n* = 58) and “Serious ADRs/ADRs mentioned in the EMA IME terms list” (45%, *n* = 59). Interestingly, only 8% (*n* = 11) stated that ADRs for medicines under additional monitoring or ADRs for new medicines should be reported.

### 3.3. Under-Reporting of Adverse Drug Reactions

**Figure 2:**
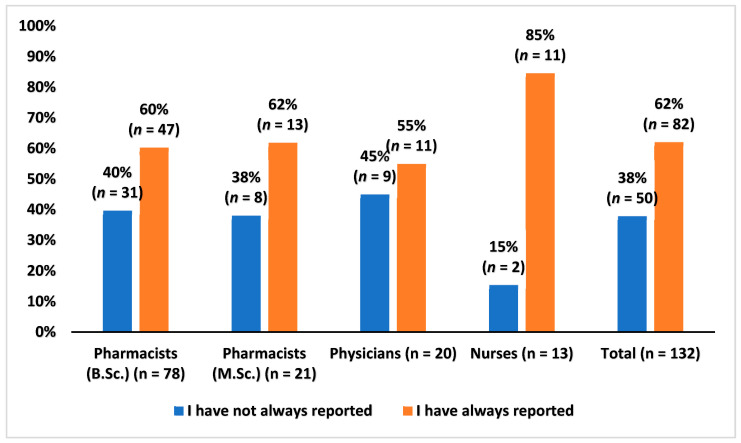
Healthcare professionals’ answer to the question of whether they had always reported suspected adverse drug reactions to the local health authority (Finnish Medicines Agency) or the marketing authorization holder when they became aware of them, percentage (%) of HCPs.

Approximately 38% (*n* = 50) of the respondents answered that they had not always reported suspected ADRs to the local health authority or the marketing authorization holder when they became aware of them. Correspondingly, 62% (*n* = 82) stated that they had always reported. The responses of different occupational groups are presented in Figure 2.

**Figure 3:**
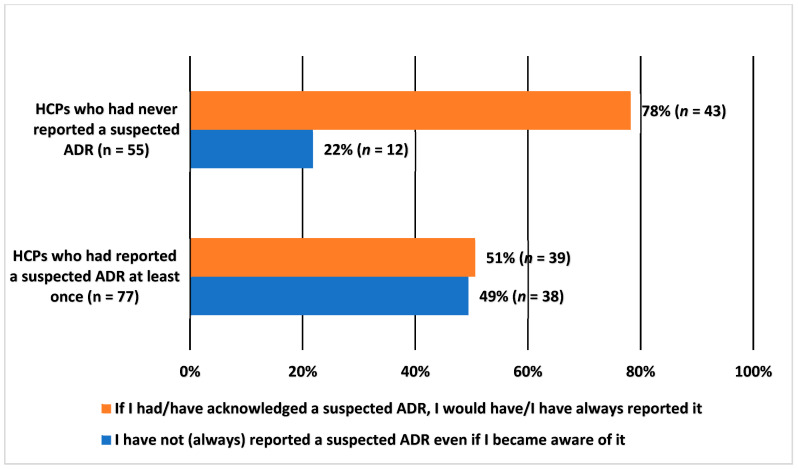
Healthcare professionals’ (HCPs) answer to the question of whether they had always reported suspected adverse drug reactions (ADRs) that came into their knowledge, percentage (%) of HCPs. Comparing the group of HCPs who had never reported ADRs with the group of HCPs who had reported at least one ADR.

When looking at the differences in the answers of HCPs who had reported at least one ADR (*n* = 77) in comparison to those who had never reported ADRs (*n* = 55), a clear difference was observed. Nearly four fifths (78%, *n* = 43) of those who had never reported ADRs answered that if they had acknowledged a suspected ADR, they would have reported it (meaning that they had never acknowledged any suspected ADRs) (Figure 3). Correspondingly, only 51% (*n* = 39) of those who had reported at least one ADR stated that they had always reported if they became aware of a suspected ADR. The difference between the answers of these two groups is statistically significant (chi-squared test, X^2^ = 10.34, df = 1, *p* = 0.001). The results were confirmed with Fisher’s exact test (*p* = 0.0018).

### 3.4. Reasons for Under-Reporting

**Figure 4:**
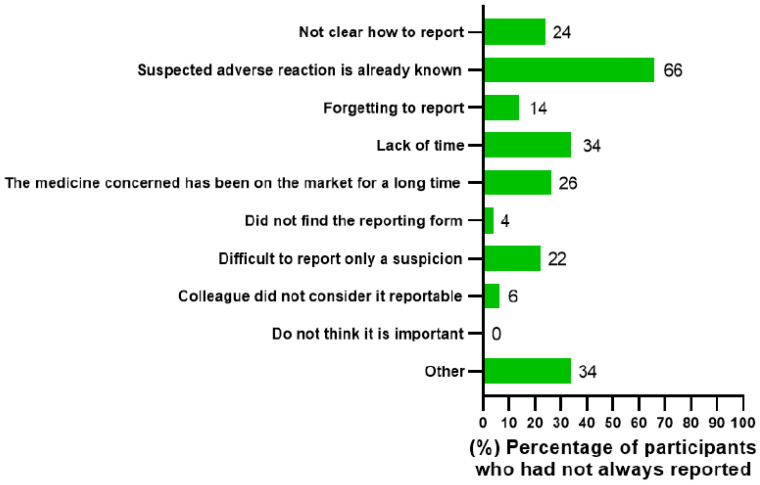
Reasons why healthcare professionals had not always reported suspected adverse drug reactions to the local health authority (Finnish Medicines Agency) or the marketing authorization holder, percentage (%) of HCPs.

Only the respondents who stated that they had not always reported suspected ADRs (*n* = 50) answered to the question regarding the reasons for under-reporting. These 50 respondents included 39 pharmacists (B.Sc. *n* = 31 and M.Sc. *n* = 8), 9 physicians, and 2 nurses. In this question, respondents were able to select as many answer options as they wanted. The most often-stated reason was that the suspected ADR is already known, which was selected by 66% (*n* = 33) of the respondents (Figure 4). The answer option “other” was selected also relatively often (34%, *n* = 17). Pharmacists, in particular, selected this answer option and in the open field they elaborated, e.g., “it is not clear what is worth reporting”, “someone else will report the ADR”, and “patient’s suspicion was not credible”.

Since most of the respondents answering this question were pharmacists, comparison between the answers of different occupational groups should be conducted with caution. However, when looking at the differences between pharmacists and physicians, it could be noted that physicians had stated lack of time as a reason for not reporting (78%, *n* = 7) more often than pharmacists (26%, *n* = 10). In fact, lack of time was the primary reason for physicians not reporting suspected ADRs.

When comparing the group of HCPs who had previously reported ADRs (*n* = 38) with the group that had never reported ADRs (*n* = 12), a noteworthy difference was observed regarding the answer option “Not clear how to report”. The majority (83%, *n* = 10) of those who had never reported had selected this answer option in comparison to only 5% (*n* = 2) of those who had reported a suspected ADR at least once.

### 3.5. Factors Actuating Adverse Drug Reaction Reporting

**Figure 5:**
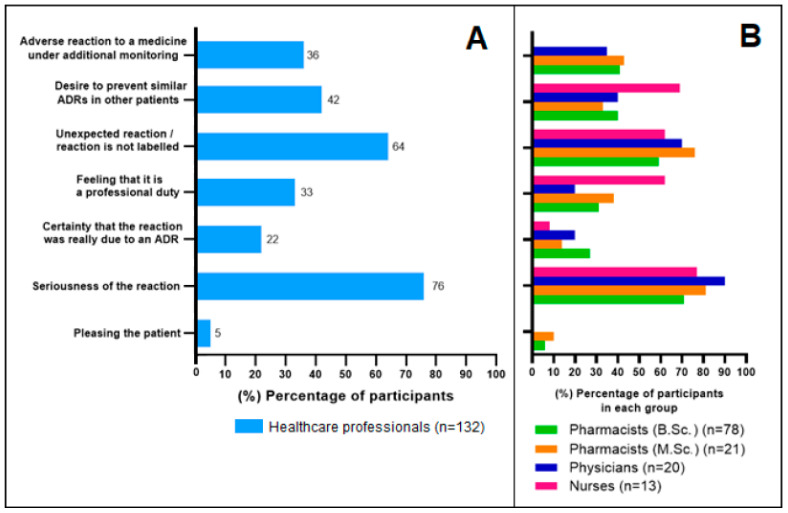
Healthcare professionals’ opinion as to what are the most motivating factors for them to report suspected adverse drug reactions (ADRs). (**A**): All responses, percentage (%) of HCPs selecting the answer option. (**B**): Percentage (%) of HCPs selecting the answer option in each occupational group.

All respondents (*n* = 132) answered the question about the factors that have motivated them, or they think would motivate them the most to report suspected ADRs. The HCPs stated that the seriousness (76%, *n* = 100) and unexpectedness (64%, *n* = 84) of the reaction are the most actuating factors (Figure 5). Many HCPs also want to report due to the desire to protect other patients from similar ADRs (42%, *n* = 55). A third of HCPs (36%, *n* = 48) see reporting as especially important for additionally monitored medicines whose safety is particularly closely monitored by the regulatory authorities.

### 3.6. Enhancing Adverse Drug Reaction Reporting

**Figure 6:**
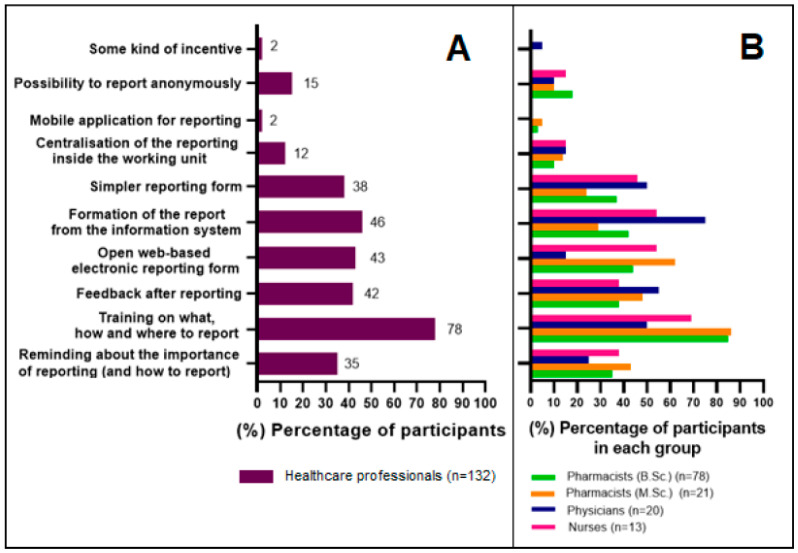
Healthcare professionals’ opinion about the most important factors that could increase the reporting of suspected adverse drug reactions. (**A**): All responses, percentage (%) of HCPs selecting the answer option. (**B**): Percentage (%) of HCPs selecting the answer option in each occupational group.

All respondents (*n* = 132) answered the question regarding the most important factors that could increase the reporting of suspected ADRs. All in all, HCPs considered “training for HCPs on what, how and where to report” as the most important factor (selected by 78%, *n* = 103 of the respondents) (Figure 6A). However, the physician subgroup regarded “formation of the report from the patient information system” even more important than training (selected by 75%, *n* = 15 of physicians) (Figure 6B). Correspondingly, physicians do not seem to see the value of having an “open web-based electronic reporting form” as this option was selected only by three physicians. Interestingly, other occupational groups seem to prefer a web-based reporting form as nearly half of them selected it. A significant share of HCPs (42%, *n* = 56) would also like to receive some feedback after reporting. No significant differences were observed between HCPs who had reported ADRs (*n* = 77) and HCPs who had not reported ADRs (*n* = 55).

#### ADR Reporting Training and Reminders

Almost half of the respondents (48%, *n* = 63) stated that they had not received any training on ADR reporting. A third of the respondents (30%, *n* = 39) had not either seen or did not remember seeing information about ADR reporting. All in all, only 16% (*n* = 21) of the respondents felt that they had received enough information about ADR reporting.

About half of the respondents (51%, *n* = 67) would prefer to get training on ADR reporting once a year. Only 8% (*n* = 11) wished to have training more frequently than once a year and 3% (*n* = 4) thought that training is not needed. The most preferred ways to receive training were an educational lecture (30%, *n* = 39), an e-learning video (29%, *n* = 38), and an e-learning educational game (23%, *n* = 30).

Respondents considered that a reminder from the patient information system used in their everyday practice (e.g., a pop-up window) would be the most effective option in reminding about ADR reporting (selected by 38%, *n* = 50). The next most effective options were considered to be reminders in the journal of the trade union (28%, *n* = 37) and reminder emails to work email address (19%, *n* = 25). Approximately one tenth (13%, *n* = 17) of the HCPs felt that there was no need for reminders.

### 3.7. Experience and Knowledge about Adverse Drug Reaction Follow-Up Requests

A quarter (25%, 19/77) of the HCPs had received follow-up (F/U) requests for additional information about the suspected ADRs that they had reported. About half of them (*n* = 10) had positive feelings about these requests as many of the HCPs felt that it is important that the health authority or marketing authorization holder has the correct understanding of the ADR. They also felt that there was a valid reason for the F/U requests they had received and that it felt good that their report had been noticed. Only two of the nineteen respondents had negative feelings about F/U requests. The ones that had negative feelings or did not know how they feel about F/U requests (*n* = 3) felt so, because they were not familiar with these requests, they did not know the answers, or they had to answer on behalf of someone else. All in all, 84% (16/19) of the HCPs stated that receiving F/U requests had not affected or could not affect their willingness to report suspected ADRs in the future. Most of the respondents (74%, 14/19) preferred to receive F/U requests via email. The current F/U process was regarded as good and only a few improvements were suggested. HCPs emphasized that the F/U requests should arrive soon after they have reported the ADRs as details are easily forgotten and it is laborious to seek the information long afterwards. Wording and layout of the requests should also be as clear as possible.

The respondents were also asked about their knowledge of why F/U requests are being sent to HCPs. Five claims were presented, and the respondents were requested to tick all the claims they considered to be correct (knowing that at least one of them was correct) (Table 2). From the three correct answer options, HCPs knew best that it is important to have as complete information as possible about certain ADRs or other reportable situations (79% answered correctly). Correspondingly, HCPs were least aware that information such as batch numbers and trade names are especially needed for biological medicinal products (32% answered correctly). Only a few HCPs thought that pharmaceutical companies try to influence HCPs with F/U requests or that sending F/U requests is mandatory in all cases. The average knowledge score for HCPs was 3.42 out of the possible 5.0.

## 4. Discussion

ADR reporting of physicians and nurses has been studied relatively extensively in other Nordic countries, but there are only a few studies concerning pharmacists. In Finland, research focusing on ADR reporting is scarce and ADR under-reporting, in particular, has not been studied at all. The Finnish ADR reporting guidance also differs from the other Nordic countries as reporting is completely voluntary for all HCP groups [12]. Our research revealed that, despite differing reporting guidance, there are still many similarities between Finnish HCPs and their Nordic counterparts when it comes to ADR reporting.

It seems that ADR reporting experience between different HCP groups in Finland has not significantly changed during the last 2 years as a similar trend can be seen compared to our previous research [30]. Nurses still have the least ADR reporting experience as 61% stated not to have reported any ADRs during their career. Similarly, this research confirms that ADR reporting is slightly more common among physicians and M.Sc. pharmacists than B.Sc. pharmacists. Based on our two studies, approximately 8–9% of HCPs report ADRs on a relatively regular basis (have reported over five ADRs during their career).

The purpose of this research was not to make an exact estimation of the amount of under-reporting in Finland as this is usually done by utilising different research methods [5,9,25]. Our research nevertheless confirms that under-reporting of ADRs also occurs in Finland. Approximately 38% of the HCPs confirmed that they had not always reported suspected ADRs when they became aware of them. It is interesting that 78% of the HCPs who have never reported an ADR, stated that they would report the ADR if they observed one. It is difficult to believe that well-experienced HCPs would never have come across any ADRs. We therefore must question the ability of HCPs to recognize ADRs and possibly think of ways to develop this ability.

In our research, the most-selected reason for under-reporting was that the suspected ADR is already known. The same finding has been discovered in Sweden in studies with physicians and nurses [26,27,28]. COVID-19 might be one of the explaining factors why the Finnish subgroup of physicians choose “Lack of time” as the primary reason (chosen by 78%) for under-reporting as it has put much pressure on physicians during the past few years. In Sweden, lack of time was only the fourth most common answer among physicians on two different occasions pre-COVID-19 [26,27]. Only 26% of the Finnish pharmacists stated that they did not have enough time to report ADRs. It is also important to notice that lack of knowledge as to how to report was the main reason why HCPs who had no ADR reporting experience did not report.

The Finnish HCPs considered seriousness of reaction as the most actuating factor (chosen by 76%) for reporting suspected ADRs, and it was followed by the unexpectedness of the reaction (chosen by 64%). These factors are also among the most important ones when considering the main purpose of the spontaneous reporting system [5]. The same factors were among the top three also in the two Swedish studies with physicians and nurses [27,28]. ADRs occurring with new drugs was the third most motivating factor in the Swedish studies. In our research it was the fourth most motivating factor after the desire to prevent similar ADRs in other patients.

In our research, HCPs felt that ADR reporting training and simplification of the reporting process (reporting directly from the patient information system, open electronic reporting form, and simpler reporting form) were the most important factors in enhancing reporting. Similar results were observed in the Swedish studies, although the portion of HCPs wanting to report directly from the patient information system was significantly lower than in Finland (12% versus 38%) [27,28].

There indeed seems to be a gap in ADR reporting training as only 52% of the HCPs had received training. Although the percentage is low, it is much higher compared to the percentage of 23% that was observed in our research pre-COVID-19 [30]. Most HCPs preferred to have training once a year or less frequently. Employees working in the pharmaceutical industry are most often given ADR reporting training annually. It might be worth considering if a similar approach would be valuable for implementing all HCPs working with patients, e.g., by utilizing modern e-learning methods. HCPs seem to be at least open to the idea as only 16% of them felt that they had received enough information about ADR reporting.

Finnish physicians felt that if the patient information system could create the ADR report directly, it would enhance reporting even more than training. There are many advantages to this approach, e.g., it could increase reporting by making it easier and faster, reduce manual errors made by HCPs, and increase the number of ADR details forwarded to pharmacovigilance databases, which in turn would make evaluation of the ADRs easier. The patient information system should also be utilized in reminding about ADR reporting according to 38% of the HCPs. Adding a notification in the system before prescribing, administering, or dispensing the medicine would ensure that the HCP is always reminded to ask about ADRs and to report them to the HA or MAH.

According to our research, 25% of the HCPs who had reported at least one ADR had received a F/U request. Only a few HCPs felt negative towards F/U requests and 84% stated that the possibility of receiving F/U requests did not affect their ADR-reporting willingness. This observation is reassuring not only for the health authorities but also for the pharmaceutical industry, which relies on F/U requests to protect the safety of the patients. Overall, HCPs thought that F/U requests are justified, and they even felt good about receiving these requests as it showed that their report had not gone unnoticed. It seems that recognition of the reports sent is important for HCPs as even 42% stated that receiving some sort of feedback of the reports would further enhance reporting. In one Swedish study, it was concluded that feedback can involve providing assessment of the causality or information about how many similar reports have been received [38].

The greatest weakness of our study is the relatively small sample size. In particular, the number of nurses and physicians recruited could have been higher to ensure that the answers from pharmacists did not overshadow answers from other groups. Therefore, the results concerning some of the subgroup analysis should be interpreted with caution. Our research is also limited by non-response bias and the fact that we could not calculate the response rates due to our questionnaire distribution method. Based on previous research, it is nevertheless expected that the response rate is low [39]. The fact that HCPs reflected their past actions and knowledge is also an important source of possible bias because answers depended on recollection and honesty. Despite these weaknesses and sources of bias, our results are aligned with previous Nordic research and can probably be extrapolated to some extent to other European countries with similar regulation and HCP educational criteria.

## 5. Conclusions

In conclusion, we observed that, as in other Nordic countries, ADR under-reporting also occurs in Finland despite the differences in reporting guidance, obligations, and routes. Based on our research, organizing recurring ADR reporting training for all HCPs working with patients could decrease under-reporting as only 52% of HCPs had received training and only 16% felt that they had enough information about reporting. Training should primarily aid in recognizing ADRs, teach how to report, and promote a reporting culture among HCPs.

ADR reporting has not become a routine for most HCPs and many want to be reminded of reporting. Information campaigns and reminders from the patient information system are effective in grasping the attention of the HCPs and keeping ADR reporting in mind while working with patients.

The collection of ADRs has improved significantly in the EU during the past decade with the introduction of the current EU pharmacovigilance legislation. For many HCPs, ADR reporting is nevertheless still a hurdle that is hard to overcome. By making ADR reporting a two-way street where HCPs also receive information in return for reporting would probably encourage reporting, enhance pharmacovigilance dialogue, and ultimately improve patient safety.

## Figures and Tables

**Table 1:** Demographics of healthcare professionals.

Profession	Physician	Nurse	B.Sc. Pharmacist	M.Sc. Pharmacist	Total
Group size (*n*)	20	13	78	21	132
Years in practice, % (*n*)					
<5	10.0 (2)	7.7 (1)	11.5 (9)	19.0 (4)	12.1 (16)
5–9	0.0 (0)	15.4 (2)	11.5 (9)	14.3 (3)	10.6 (14)
10–19	15.0 (3)	23.1 (3)	37.2 (29)	38.1 (8)	32.6 (43)
>20	75.0 (15)	53.8 (7)	39.7 (31)	28.6 (6)	44.7 (59)
Primary workplace, % (*n*)					
Healthcare center	45.0 (9)	69.2 (9)	1.3 (1)	0.0 (0)	14.4 (19)
Hospital	35.0 (7)	15.4 (2)	10.3 (8)	9.5 (2)	14.4 (19)
Retail pharmacy	0.0 (0)	0.0 (0)	83.3 (65)	66.7 (14)	59.8 (79)
Private clinic	20.0 (4)	0.0 (0)	0.0 (0)	0.0 (0)	3.0 (4)
Hospital pharmacy	0.0 (0)	0.0 (0)	3.8 (3)	23.8 (5)	6.1 (8)
Other	0.0 (0)	15.4 (2)	1.3 (1)	0.0 (0)	2.3 (3)

Background information reported by the respondents.

**Table 2:** Follow-up request knowledge among Finnish HCPs, % correct, (*n*/*n*) correct answers/all answers.

Claim ^a^	All HCPs (*n* = 19)
The original report is incomplete (yes)	47.4% (9/19)
It is important to have as complete information as possible about certain ADRs or other reportable situations related to the use of the medicinal product (e.g., use during pregnancy) (yes)	78.9% (15/19)
In the case of biological medicinal products in particular, it is important to obtain certain information (such as batch number and trade name) about the medicinal product (yes)	31.6% (6/19)
Pharmaceutical companies want to influence HCPs’ opinion about a medicine when a potential ADR has occurred (no)	94.7% (18/19)
It is mandatory for pharmaceutical companies to send F/U requests (requests for additional information) in all cases (no)	89.5% (17/19)
Total number of right answers	68.4% (65/95)
Average knowledge score per responder	3.42 (65/19)

HCP healthcare professional, ADR adverse drug reaction, F/U follow-up. ^a^ Correct answer is presented in brackets after the claim.

## Data Availability

Data is not available online.

## Supplementary Materials

The following supporting information can be downloaded at: https://www.mdpi.com/article/10.3390/healthcare10061015/s1, Questionnaire File.
