# Supplementary material for: Under-Reporting of Adverse Drug Reactions in Finland and Healthcare Professionals’ Perspectives on How to Improve Reporting
Source: Healthcare (Basel). 2022 May 31;10(6):1015. doi: 10.3390/healthcare10061015 (PMC9222550; doi:10.3390/healthcare10061015)
Supplement: Supplementary file 1 [file healthcare-10-01015-s001.zip › healthcare-1709638-supplementary.pdf]

Dear healthcare professional,

You're welcome to participate to this questionnaire about the improvement of adverse drug reaction reporting in Finland. Participants answer the questionnaire anonymously and it takes about 10 to 20 minutes to fill in the questionnaire form (depending on your responses).

It is voluntary to participate to the questionnaire and by answering the questionnaire you are giving an informed consent to participate in the research. If you wish, you can stop answering the questionnaire at any time before it ends.

In this questionnaire, all adverse events that occur during the use of a medicinal product and are spontaneously reported by healthcare professionals are counted to be suspected adverse drug reactions (even if the causal relationship is unknown).

Please do not hesitate to contact me if you have any questions about the questionnaire or the research I'm conducting. In case you decide to participate in this research, please answer to the questionnaire by xx.xx.2021.

With respect,
Veera Salminen
M.Sc. Pharmacy student
University of Helsinki
veera.salminen@helsinki.fi

Responsible researcher:
Mia Sivén
PhD Pharmacy, Docent
University of Helsinki
mia.siven@helsinki.fi

Background information

1. What is your occupation?
a) Physician
b) Specialized physician
c) Dentist
d) Nurse (registered)
e) Practical nurse
f) Pharmacist (M.Sc. Pharm.)
g) Pharmacist (B.Sc. Pharm.)
h) Other, what?

2. How many years have you worked in your profession?
a) less than 5 years
b) 5-9 years
c) 10-19 years
d) 20 years or more

3. What is your main working place?
a) Health center
b) Hospital
c) Pharmacy
d) Private clinic
e) Hospital pharmacy
f) Pharmaceutical industry
g) Government
h) Other, what?

Adverse drug reaction reporting - background

4. During your career, how many times have you reported about suspected adverse drug reactions to the Finnish Medicines Agency or the marketing authorisation holder?
a) Never
b) 1-5 times
c) 6-10 times
d) More than 10 times

5. Answer according to your current understanding. In Finland, healthcare professionals are obliged to report suspected adverse drug reactions to the Finnish Medicines Agency or the marketing authorisation holder.

a) Yes

b) Adverse drug reaction reporting is only obligatory when the concerned medicine is under additional monitoring (having black triangle)

c) Adverse drug reaction reporting is only obligatory when the concerned medicine is a vaccine

d) No

e) I do not know

6. In your opinion, what kind of adverse drug reactions should be reported (describe in few words)?

7. Have you ever acknowledged a suspected adverse drug reaction but have not reported it to the Finnish Medicines Agency or the marketing authorisation holder of the medicinal product?

a) Yes

b) No (by selecting this option you will next move to question 9)

<u>Reasons for under-reporting</u>

8. Why did you not report a suspected adverse drug reaction to the Finnish Medicines Agency or the marketing authorisation holder of the medicinal product (you may select multiple options)?

a) It is not clear for me how the reporting should be done

b) The suspected adverse drug reaction is already known

c) I forgot

d) There is not enough time for reporting

e) The medicinal product has been on the market for a long time already

f) I did not find the reporting form

g) It is hard to report only a suspicion

h) My colleague did not think it was necessary to report

i) I do not find it important

j) Other, what?

Motivating factors

9. Which factors motivate you the most to report suspected adverse drug reactions (you may choose 1-3 most important factors to you)? If you have never reported, which factors do you think would affect the most to your willingness to report?
a) Pleasing the patient
b) Seriousness of the adverse drug reaction
c) Certainty that the adverse drug reaction is associated to the medicinal product
d) Feeling that it is a professional duty
e) Unexpected reaction / reaction is not labelled (cannot be found from the product information of the medicinal product)
f) Desire to prevent similar adverse drug reactions in other patients
g) Adverse drug reaction to a new medicine under additional monitoring (having the black triangle)
Other, what?

Increasing the number of reports

10. Which of the following factors do you consider as most important factors that could increase the number of relevant adverse drug reaction reports by Finnish healthcare professionals (you may choose 1-4 most important factors to you)?
a) Reminding about the importance of reporting (and how to report) every now and then e.g. by email
b) Training for healthcare professionals on what, how and where to report suspected adverse drug reactions
c) Feedback after reporting (e.g. getting the medical evaluation done to the report 'for information')
d) Making it possible to report electronically also without Fimnet login details (open web-based electronic reporting form)
e) Making it possible to report straight from the patient's records without a separate reporting form (e.g. formation of the report from the information system, taking data protection into account)
f) Making the reporting form more simple
g) Centralisation of the reporting inside the working unit to a healthcare professional who has been trained for reporting
h) Taking a mobile application into use for reporting purposes
i) Possibility to report anonymously
j) Some kind of incentive for reporting, what?

11. Do you have any other suggestions that could increase the reporting of relevant suspected adverse drug reactions by healthcare professionals in Finland? (voluntary question)

12. What do you think would be the most suitable way to remind about reporting of adverse drug reactions every now and then?
a) I do not think there is a need for a reminder
b) General reminder from the information system in use (e.g. as a pop-up window)
c) News or an article in the member journal of a trade union
d) General email reminder to my work email address
e) Other, what?

13. Where have you got information about reporting of adverse drug reactions (e.g. some organization's website, training material, product information of medicinal products, studies)? Enter "I have not received any information" if you have not received or do not remember receiving information about the subject.

14. Do you think you have received enough information about reporting of adverse drug reactions?
a) Yes
b) No
c) I have not received or do not remember receiving information about reporting of adverse drug reactions

15. Which organizations have given you training about reporting of adverse drug reactions (you may choose one or more options)?
a) I have not received training on adverse drug reaction reporting
b) University / university of applied sciences during studies
c) Trade union
d) The Finnish Medicines Agency Fimea
e) Marketing authorisation holders / pharmaceutical companies
f) Pharma Industry Finland
g) External service provider
h) Internal trainer
i) Other organization, what?

16. How often would you like to have training on adverse drug reaction reporting?
a) I do not think training is necessary
b) Less than once a year
c) Once a year
d) 2-3 times a year
e) 4 times a year
f) More than 4 times a year

17. How would you prefer the training to be organized?
a) I do not think training is necessary
b) E-learning educational game related to adverse drug reactions and reporting
c) E-learning video related to adverse drug reactions and reporting
d) Workshop-type training session
e) Educational lecture
f) Other, what?

<u>Follow-up requests - 1</u>

18. Have you received follow-up requests (requests for additional information) of the suspected adverse drug reactions that you have reported?
a) Yes
b) No (by selecting this you will next move to question 25)
c) I have never reported a suspected adverse drug reaction (by selecting this you will next move to question 25)

<u>Follow-up requests - 2</u>

19. How do you react to follow-up requests of adverse drug reactions?
a) Positively
b) Negatively
c) Neutrally
d) I do not know / cannot say
e) Other, what?

20. Why are you reacting to follow-up requests in a before mentioned way?

21. Why do pharmaceutical companies send follow-up requests (choose the options you consider to be correct, there may be one or more correct options)?
a) The original report is incomplete
b) Pharmaceutical companies want to influence healthcare professionals' opinion about a medicine when a potential adverse reaction has occurred
c) It is important to have as complete information as possible about certain adverse drug reactions or other reportable situations related to the use of the medicinal product (e.g. use during pregnancy)
d) In the case of biological medicinal products in particular, it is important to obtain certain information (such as batch number and trade name) of the medicinal product
e) It is mandatory for pharmaceutical companies to send follow-up requests (requests for additional information) in all cases

22. How would you like to receive follow-up requests?
a) I do not want to receive follow-up requests
b) Via mobile application
c) By phone
d) By email
e) By letter (regular mail)
f) Other, what?

23. Would you have any suggestions how the follow-up process could be done as easy as possible for you or how it could be improved? (voluntary question)

24. Do you think that receiving follow-up requests could affect or has affected negatively on your willingness to report suspected adverse drug reactions in future?
a) Yes, why?
b) No

25. Any other possible comments or observations related to reporting of adverse drug reactions, follow-up requests or this questionnaire/research. (voluntary question)

Many thanks for your participation!
